# Risk factors and cardio-metabolic outcomes associated with metabolic-associated fatty liver disease in childhood

**DOI:** 10.1016/j.eclinm.2023.102248

**Published:** 2023-10-06

**Authors:** Jasmin de Groot, Susana Santos, Madelon L. Geurtsen, Janine F. Felix, Vincent W.V. Jaddoe

**Affiliations:** a The Generation R Study Group, Erasmus University Medical Center, Rotterdam, the Netherlands; b Department of Pediatrics, Erasmus University Medical Center, Rotterdam, the Netherlands; c EPIUnit, Instituto de Saúde Pública, Universidade do Porto, Porto, Portugal; d Laboratório para a Investigação Integrativa e Translacional em Saúde Populacional (ITR), Universidade do Porto, Porto, Portugal

**Keywords:** Metabolic-associated fatty liver disease, Pediatric liver fat, Pediatric metabolic health, Pediatric obesity, Liver fat, Cardio-metabolic health

## Abstract

**Background:**

Non-Alcoholic Fatty Liver Disease (NAFLD) is defined as increased liver fat percentage, and is the most common chronic liver disease in children. Rather than NAFLD, Metabolic-Associated Fatty Liver Disease (MAFLD), defined as increased liver fat with presence of adverse cardio-metabolic measures, might have more clinical relevance in children. We assessed the prevalence, risk-factors and cardio-metabolic outcomes of MAFLD at school-age.

**Methods:**

This cross-sectional analysis was embedded in an ongoing population-based prospective cohort study started in 2001, in the Netherlands. In 1910 children of 10 years, we measured liver fat fraction by magnetic resonance imaging (MRI), body mass index (BMI), blood pressure, and lipids, insulin, and glucose concentrations. Childhood lifestyle factors were obtained through questionnaires. MAFLD was defined as ≥2% liver fat in addition to excess adiposity (BMI or visceral adiposity), presence of metabolic risk (blood pressure, triglycerides and HDL-concentrations) or prediabetes (glucose).

**Findings:**

Of all children, 49.6% had ≥2% liver fat, and 25.2% fulfilled the criteria of MAFLD. Only non-European descent was associated with increased odds of MAFLD at nominal significance (Odds Ratio 1.38, 95% Confidence Interval 1.04, 1.82). Compared to children with <2% liver fat, those with MAFLD had increased odds of cardio-metabolic-risk-factor clustering (Odds Ratio 7.65, 95% Confidence Interval 5.04, 11.62).

**Interpretation:**

In this study, no NAFLD-associated childhood risk factors were associated with increased odds of childhood MAFLD, yet the findings suggest that ethnicity could be, despite mostly explained by socio-economic factors. Use of MAFLD criteria, rather than NAFLD, may identify children at risk for impaired cardio-metabolic health.

**Funding:**

Erasmus University MC, the 10.13039/501100001826Netherlands Organisation for Health Research and Development, the Ministry of Health, Welfare, and Sport, and the 10.13039/501100000781European Research Council.

**Research in context**

**Evidence before this study**

Non-Alcoholic Fatty Liver Disease (NAFLD) is a condition in which liver cells have increased fat deposition in the absence of genetic or metabolic diseases or other directly identifiable external causes, such as medication use, substance abuse or malnutrition. Due to the nature of this diagnosis, an expert panel introduced a redefinition in 2021, namely Metabolic-Associated Fatty Liver Disease (MAFLD), a diagnosis with a set of different inclusion criteria. We searched Pubmed using the terms ‘[“Metabolic-Associated Fatty Liver Disease” AND “pediatric”] as well as just [“Metabolic Associated Fatty Liver Disease”], with no language or time restrictions. We found that in existing MAFLD literature, this definition has mostly been applied in clinically obese adult populations, and has, thus far, shown to be useful in cardio-metabolic risk stratification in adults. When specifying the search to a pediatric population, the number of articles decreased from over 600 results to 24 results. Only a few studies analyzed MAFLD in relation to determinants and cardio-metabolic health outcomes in children, but mostly in obese populations.

**Added value of this study**

We assessed the prevalence, early-life risk factors and cardio-metabolic outcomes associated with MAFLD in childhood, in a general pediatric population. This study was embedded in a large population-based cohort study, with a relatively large sample size, and Magnetic Resonance Imaging (MRI) estimated liver fat percentages. This allowed us to more accurately estimate liver fat fraction in a non-clinical population, something that has not yet been done for this set of diagnostic criteria. Furthermore, this study was able to take many childhood factors as well as cardio-metabolic outcomes into consideration, in order to analyze adverse cardio-metabolic profiles in relation to MAFLD in childhood.

**Implications of all the available evidence**

Our findings suggest that, in a general pediatric population, none of the childhood factors previously associated with NAFLD appear to have a strong association with MAFLD after correcting for socio-economic factors. However, a nominal association of ethnic background with MAFLD was found, and further exploration of specific ethnic groups suggests differences in association between the ethnic subgroups. Use of the MAFLD definition might contribute to the identification of children at risk for adverse cardio-metabolic risk profiles and potential later life health consequences.

## Introduction

Pediatric Non-Alcoholic Fatty Liver Disease (NAFLD) is the most common cause of chronic liver disease in children worldwide, with an estimated prevalence of 8% in general populations and 34% in obese pediatric populations.^1^ NAFLD is associated with cardio-metabolic disease, hepatocellular carcinoma and end-stage liver disease, and is the most common cause for liver transplantation in both children and adults.^2^^,^^3^ NAFLD is normally defined by a liver fat fraction of ≥5% in absence of other causes that may lead to liver steatosis, such as genetic or metabolic disorders, infections, use of medications, alcohol consumption, or malnutrition.^4^ Partly due to its exclusionary nature, this definition leads to a diagnosis often accompanied by excessive testing in children.^5^ In 2021, it was suggested to redefine pediatric NAFLD, as a diagnosis of exclusion, into pediatric Metabolic-Associated Fatty Liver Disease (MAFLD), with a set of inclusion criteria.^6^^,^^7^ In this concept, pediatric MAFLD is a subtype of pediatric fatty liver disease, diagnosed by the presence of hepatic steatosis in addition to at least one of three criteria: excess adiposity, presence of metabolic risk factors or prediabetes.^6^ These criteria were specifically selected to highlight the role of cardio-metabolic dysregulation in hepatic steatosis patients, as there is increasing evidence of elevated cardiovascular and metabolic disease risk in this population.^8^ Previous studies in adults have shown that MAFLD criteria aid in identifying patients with significant hepatic fibrosis, incident cardiovascular disease, and chronic kidney disease.^9^^,^^10^ There is accumulating evidence for the validity of using the concept of MAFLD, instead of NAFLD, for better risk stratification and clinical practice in adults.^10^^,^^11^ However, in pediatric populations, the prevalence of MAFLD and its cardio-metabolic consequences are largely unknown.

In a population-based study among 1910 children aged 10 years in Rotterdam, the Netherlands, we aimed to estimate the prevalence and to identify risk factors and cardio-metabolic outcomes of MAFLD in childhood. We assessed liver fat by magnetic resonance imaging.

## Methods

### Study population

**Fig. 1:**
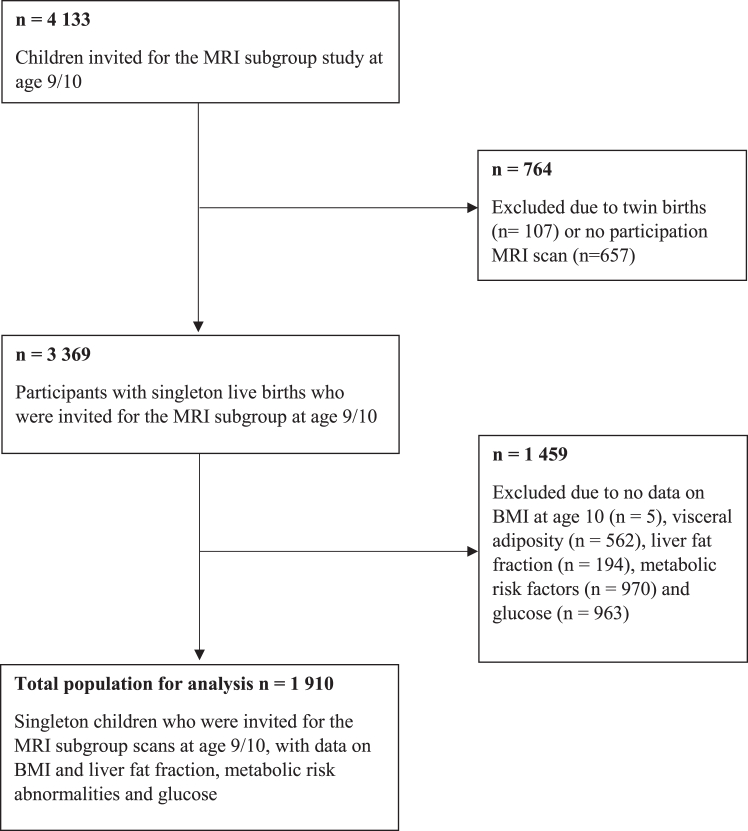
Flowchart.

This cross-sectional study was embedded within the Generation R Study, a population-based prospective cohort from early fetal life onward, set in Rotterdam, the Netherlands.^12^ All children were born between April 2002 and January 2006. In total, 4133 children were invited to the MRI subgroup study at 10 years. None of these children had been diagnosed with type 2 diabetes. Singleton children who underwent an MRI scan at 10 years, with no missing information on any of the other criteria for MAFLD were included, leading to a final study sample of 1910 school-aged children (Fig. 1).

### Ethics statement

The Generation R study has been approved by the Medical Ethical Committee of the Erasmus MC, University Medical Center Rotterdam (MEC 198.782/2001/31). Written informed consent was obtained for all participants.^12^

### Childhood liver fat and MAFLD

At a median age of 10.0 (95% range 9.5, 11.9 years) years, liver fat was measured using a 3.0 T MRI scanner (Discovery MR750w, GE Healthcare, Milwaukee, Wisconsin, United States).^2^^,^^12^ Children wore light clothing without metal objects. A liver fat scan was performed using a single-breath-hold, 3D volume and special 3-point proton density weighted Dixon technique (IDEAL IQ), producing 50 image slices of 5 mm thickness.^13^ The obtained fat-fraction maps were analyzed by Precision Image Analysis (PIA, Kirkland, Washington, United States) using the sliceOmatic (TomoVision, Magog, Canada) software package.^2^^,^^14^ All extraneous structures and any image artifacts were removed manually.^14^ Liver fat fraction was assessed from four regions of interest of at least 4 cm^2^, which were manually drawn from different slices from the central portion of the hepatic volume. Subsequently, the mean signal intensities were averaged to generate an overall mean liver fat estimation. This was done by different observers who were each assigned different portions of the data. All observers were trained to the same protocols and standards of measurement. Liver fat measured with IDEAL IQ (General Electrics, Boston, United States) using MRI is reproducible, highly precise and validated in adults.^15^ None of these children were known to have been diagnosed with any liver pathology which was asked through questionnaires and during visits every 4 years.^12^

For the definition of MAFLD, two main criteria must be met: evidence of hepatic steatosis, and evidence of metabolic risk.^6^ The specific criteria, as originally proposed, are given in Table 1.^6^ Due to our relatively healthy study sample and available data, some modifications to the criteria were made for this study. A recent study from the same cohort as the current study showed that a 2% liver fat fraction is already associated with an adverse cardio-metabolic risk profile at 10 years.^2^ Furthermore, there is currently no consensus on the appropriate liver fat fraction cut-off using MRI imaging in children.^16^ In the current study, we applied the cut-off of 2% liver fat fraction measured by MRI, as observed in our cohort previously, and used the 5% liver fat fraction cut-off for a sensitivity analysis. For the second criteria for MAFLD, we made two modifications. First, since we did not have waist circumference data, we used visceral adiposity, applying a 90th percentile cut-off. Second, since we only had non-fasting blood samples available and no children who were diagnosed with type 2 diabetes, a child was defined as at risk for prediabetes if their non-fasting serum glucose was above the 90th percentile in our cohort.^17^

**Table 1:** Diagnostic criteria of MAFLD and the application in the current study.

	Expert panel definitions	Application in current study
**1st Criterion—Hepatic steatosis**		
Evidence of hepatic steatosis	An estimated ≥5% liver fat fraction, based on outcome of a liver biopsy, imaging (MRI or echo) or blood biomarkers.	Applied 2 different cut-offs: - ≥2% liver fat fraction estimated from MRI scans. - ≥5% liver fat fraction estimated from MRI scans.
**2nd Criterion—Metabolic risk factor (at least one)**		
Excess adiposity	Either: - a BMI ≥1 SD of the WHO growth reference median^5^ - waist circumference ≥90th percentile^2^	Either: - a BMI ≥1 SD of the WHO growth reference median - visceral adiposity (measured by MRI) ≥90th percentile.
Normal weight with metabolic dysregulation	A normal weight (between −1 and +1 SD of the WHO reference median) and 2 or more of the following: - Plasma triglyceride ≥90th percentile - Systolic and/or Diastolic blood pressure ≥90th percentile - HDL-cholesterol <10th percentile - Triglyceride:HDL ratio ≥2.25	A normal weight (between −1 and +1 SD of the WHO reference median) and 2 or more of the following: - Plasma triglyceride ≥90th percentile - Systolic and/or Diastolic blood pressure ≥90th percentile - HDL-cholesterol <10th percentile - Triglyceride:HDL ratio ≥2.25
(Pre)diabetes mellitus type 2	According to international diagnostic criteria: Type 2 diabetes (at least 1 of 3): 1) Fasting serum glucose ≥126 mg/dL; 2) HbA1c ≥ 6.5%; or 3) Existing clinical diagnosis Prediabetes (at least 1 of 2) (ref): 1) Fasting serum glucose between 100 mg/dL and 12 mg/dL; 2) HbA1c ≥ 5.7% and <6.5%	Risk for prediabetes defined as: - ≥90th percentile non-fasting serum glucose^6^

BMI: body mass index; DBP: diastolic blood pressure; HbA1c: hemoglobin A1c; HDL: high-density lipoprotein; MRI: magnetic resonance imaging; SD: standard deviation; WHO: World Health Organization.

### Childhood risk factors

In current literature, (pediatric) NAFLD is often used interchangeably with liver fat accumulation, and the MAFLD criteria appear to be too recent for the publication of studies analyzing MAFLD-specific risk factors. Therefore, potential childhood risk factors for MAFLD considered in this study were selected based on their previously reported associations with liver fat accumulation and NAFLD.^1^^,^^2^^,^^18^ From medical records we obtained sex, gestational age at birth, and birth weight.^12^ Sex- and gestational age-adjusted birth weight standard deviation scores (SDS) were calculated based on growth curves developed by Niklasson et al.^19^ Months of breastfeeding, sugar intake at 1 years old (in servings), average screen-time per day (≥2 h; <2 h), and exercise per day (≥2 h; <2 h) at 10 years old, were obtained through repeated questionnaires completed by the primary caregiver.^12^ Ethnicity was obtained from questionnaires, where a child was considered of Dutch background if both parents were born in the Netherlands, and of non-Dutch origin if one or both of the parents were born abroad. If the parents were born in different countries, the country of birth of the mother determined the ethnic background of the child. This definition is derived from the Central Bureau of Statistics Netherlands.^20^ We identified the largest ethnicity groups in our sample, which were Dutch/European, Cape Verdean, Dutch Antillean, Moroccan, Surinamese-Creole and Surinamese-Hindustani and Turkish children.

### Childhood cardio-metabolic outcomes

At the age of 10 years, we calculated BMI from the height and weight of the children, which were measured without shoes and heavy clothing. Subsequently, we calculated the sex- and age-adjusted SDS of childhood BMI based on WHO reference growth charts (Growth Analyzer 4.0, Dutch Growth Research Foundation).^21^ Childhood BMI cut-offs to categorize overweight and obesity were ≥1 SD and ≥2 SD, respectively.^21^ Visceral fat mass was measured using MRI as described previously.^2^^,^^14^ Blood pressure was measured, while sitting, at the right brachial artery three times with 1-min intervals using the validated automatic sphygmomanometer Datascope Accutor Plus (Paramus, New Jersey, United States).^22^ We calculated mean blood pressure at 10 years using the last three blood pressure measurements of each participant. We collected 30-min fasting venous blood samples at 10 years, hereafter referred to as non-fasting samples. We measured glucose, HDL-cholesterol, and triglyceride concentrations using the c702 module on the Cobas 8000 analyzer and insulin concentrations using the electrochemiluminescence immunoassay on the E411 module (Roche, Almere, the Netherlands).^12^ From the total and HDL-cholesterol concentrations, we estimated the serum LDL-concentrations using the Friedewald formula.^23^ We defined clustering of cardio-metabolic risk factors as having three or more out of the following four adverse risk factors: visceral fat mass above the 75th percentile; systolic or diastolic blood pressure above the 75th percentile; HDL-cholesterol below the 25th percentile or triglycerides above the 75th percentile; and insulin above the 75th percentile of our study.^2^ The percentile cut-off values for cardio-metabolic clustering outcomes as well as the MAFLD criteria in this study sample can be found in Supplementary Table S1.

### Covariates

The Directed-Acyclic-Graphs (DAGs) of the potential confounder relationships for the analyses can be found in Supplementary Fig. S1A and B. Parental factors included the continuous variables maternal age at intake and maternal pre-pregnancy BMI, and the categorical variables maternal highest education finished (higher education, no higher education), parity (multiparity, nulliparity), living situation of parents (living together, not living together), and household income per month (<2000 euros, 2000–4000 euros, >4000 euros). Pregnancy factors included any smoking and alcohol use during pregnancy, all of which were obtained through repeated questionnaires completed by the mothers in each trimester of pregnancy.^12^ Information on pregnancy complications (gestational hypertension or pre-eclampsia) was obtained from medical records.

### Statistical analyses

First, we performed a non-response analysis by comparing children included and excluded in the analyses with ANOVA and Chi-square tests for continuous and categorical variables, respectively. Second, we assessed the associations of known determinants of NAFLD in childhood, (sex, ethnicity, birth weight, gestational age at birth, months of breastfeeding, sugar intake at infancy, screen-time and exercise at 10 years) with the odds of having ≥2% liver fat with or without MAFLD by using multinomial regression analyses. The basic models were adjusted only for sex and age at MRI, the determinant models were additionally mutually adjusted for the other potential childhood determinants, and the final models were additionally adjusted for maternal education and household income. Due to the small numbers for specific ethnic groups, we performed the main analyses with ethnicity as a dichotomous variable (European, Non-European). We performed an additional explorative analysis with ethnicity as a 7-category variable, with groups of Dutch/European, Cape Verdean, Dutch Antillean, Moroccan, Surinamese-Creole, Surinamese-Hindustani, and Turkish children. Third, we assessed associations of liver fat and MAFLD with cardio-metabolic risk factors and clustering by linear and logistic regression analyses. For these analyses, children were categorized as liver fat fraction <2%; liver fat fraction ≥2% without MAFLD; and liver fat fraction ≥2% with MAFLD. We compared children with liver fat fraction ≥2% without MAFLD to those with liver fat fraction ≥2% with MAFLD in separate regression models. We adjusted the basic models were for age at MRI only, and the final models additionally for potential confounders (sex, ethnicity, maternal age, education, pre-pregnancy BMI, alcohol use during pregnancy, smoking during pregnancy, and pregnancy complications, parental living situation, household income, gestational age at birth, breastfeeding months, sugar intake in infancy, and hours of exercise per day and screen-time per day at 10 years). Confounders were considered for the analyses based on previous associations observed with exposures and liver fat accumulation, and were included based on effect estimate changes of ≥10%, calculated through: % change = ((β2- β1)/β1) ∗ 100. Assumptions for each analysis were checked prior to conducting them, and all the main analyses were corrected for multiple testing by controlling for a False Discovery Rate (FDR) of 0.05. Missing data in the risk factors and covariates were imputed using the Markov Chain Monte Carlo approach, additionally using the following variables as indicators for imputation: caloric intake during pregnancy, diet score during pregnancy, age of the mother at intake, BMI of the partner, education level of the partner, and child serum insulin at 10 years. We assumed a missing at random (MAR) structure, based on the missingness patterns observed. Fifty imputed datasets were created and analyzed for all analyses. Supplementary Table S2 gives an overview of the percentages missing per variable. All analyses were performed using IBM SPSS Statistics 25.0 for Windows.

### Role of the funding source

The funding sources did not have any role in the study design, analysis, interpretation, or writing of this manuscript, nor any role it the decision to publish. J.G., S.S. and V.W.V.J had access to the data, and J.G. and V.W.V. J. had final responsibility for the decision to submit for publication.

## Results

### Subject characteristics

The median liver fat percentage was 1.99% (95% range 1.23, 4.93). In total, 18.4% (n = 351) of all children had overweight, and 6.0% (n = 114) had obesity. Of all children, 49.6% (n = 947) had ≥2% liver fat, and 25.2% (n = 481) fulfilled the criteria of MAFLD using a ≥2% cut-off value (Table 2). The prevalence of MAFLD was 69.2% among children with overweight, and 82.5% among children with obesity. Within the MAFLD population, 50.5% children had overweight, and 19.5% of these children had obesity. Supplementary Table S3 gives an overview of the subject characteristics per liver fat group. When using the 5% liver fat cut-off, 2.2% (n = 42) of the population met the MAFLD criteria (Supplementary Table S4). Non-response analyses indicated that as compared to children included in our study sample, those not included had slightly more frequently obesity, preterm birth and lower household income, and less mothers finished a higher education (Supplementary Table S5).

**Table 2:** Population characteristics.

	Total population (n = 1910)
Maternal pregnancy characteristics	
Age at intake (years)	31.2 (4.7)
Higher education (%)	54.0 (957)
Parents living together (%)	89.6 (1578)
Household income at 5 years (%)	
<2000 euros	18.7 (299)
2000–4000 euros	43.7 (701)
>4000 euros	37.6 (603)
Multiparity (% yes)	43.1 (796)
Pre-pregnancy body mass index (kg/m^2^)	22.6 (18.1, 35.2)
Any alcohol use during pregnancy (% yes)	59.4 (901)
Any smoking during pregnancy (% yes)	21.3 (354)
Pre-eclampsia (% yes)	1.9 (30)
Gestational hypertension (% yes)	3.4 (56)
Child characteristics	
Female (%)	51.6 (989)
Ethnicity (%)	
European	69.1 (1292)
Non-European	30.9 (579)
Birth weight (grams)	3475 (553)
Gestational age at birth (weeks)	40.1 (35.9, 42.3)
Preterm birth (%)	3.9 (74)
Breastfeeding duration (months)	3.5 (0.0, 12.0)
Sugar intake at 12 months (servings/day)	1.57 (0.4, 4.8)
Less than 2 h of screen-time/day at 10 years (%)	49.1 (721)
More than 2 h of exercise/day at 10 years (%)	22.7 (359)
Adiposity and cardiovascular outcomes	
Age at assessment (years)	10.0 (9.5, 11.9)
MAFLD group (%)	
<2% liver fat	50.4 (963)
≥2% liver fat no MAFLD	24.4 (466)
MAFLD	25.2 (481)
Liver fat fraction (percentage)	1.99 (1.23, 4.93)
Body mass index, (SDS)	0.32 (1.03)
Overweight (%)	18.4 (351)
Obesity (%)	6.0 (114)
Visceral adiposity (g/cm^3^)	125.0 (56.7, 321.7)
Systolic blood pressure (mmHg)	103.2 (7.8)
Diastolic blood pressure (mmHg)	58.5 (6.4)
Glucose (mmol/L)	5.27 (0.93)
Insulin (pmol/L)	178 (35, 613)
Triglycerides (mmol/L)	0.95 (0.41, 2.59)
HDL-cholesterol (mmol/L)	1.50 (0.34)
LDL-cholesterol (mmol/L)	2.81 (0.65)
Ratio triglycerides/HDL-cholesterol	0.65 (0.22, 2.36)

Note: values are observed, not imputed data and represent means (SD), medians (95% range) or valid % (n) unless otherwise stated. The mean and standard deviation are given for all normally distributed continuous variables, and the median and 95% range for non-normally distributed variables.

MAFLD: metabolic-associated fatty liver disease; SD: standard deviation; HDL: high-density lipoprotein; LDL: low-density lipoprotein.

### Childhood risk factors, liver fat and MAFLD

Results from the basic and the determinant models showed that non-European descent, fewer months of breastfeeding and screen-time ≥2 h/day at 10 years were associated with MAFLD at school age (all p-values < 0.05 without FDR correction) (Supplementary Tables S6 and S7). In the final model, only non-European descent had higher odds of MAFLD (OR 1.38, 95% CI 1.04, 1.82), compared to children of European descent, although this association was not significant after FDR-correction. None of the other childhood characteristics were associated with a difference in odds between those with <2% liver fat and those with ≥2% liver fat without MAFLD after adjustment for socio-economic variables (Table 3). The exploratory analysis focused on the ethnic differences showed that in the full model higher odds ratios for MAFLD were only observed in children of Turkish descent (OR 2.50, 95% CI 1.50, 4.16) (Supplementary Table S8). When comparing the MAFLD group to those with ≥2% liver fat without MAFLD, we observed that Non-European ethnicity, compared to European ethnicity, was associated with higher odds of MAFLD (OR 1.38, 95% CI 1.01, 1.90) (Supplementary Table S9).

**Table 3:** Childhood risk factors for increased liver fat and MAFLD (n = 1910).

	<2% Liver Fat	≥2% Liver Fat without Metabolic-Associated Fatty Liver Disease	≥2% Liver Fat with Metabolic-Associated Fatty Liver Disease
	Odds Ratio (95% Confidence Interval)		
Childhood characteristics			
Sex	Reference	..	..
Male	..	Reference category	Reference category
Female	..	1.01 (0.81, 1.27)	1.11 (0.89, 1.40)
Ethnicity	Reference	..	..
European	..	Reference category	Reference category
Non-European	..	0.99 (0.74, 1.33)	1.38 (1.04, 1.82)
Birth weight, SDS	Reference	1.05 (0.94, 1.17)	1.01 (0.91, 1.13)
Gestational age, weeks	Reference	1.00 (0.94, 1.07)	0.99 (0.92, 1.05)
Breastfeeding, months	Reference	0.98 (0.95, 1.02)	0.97 (0.93, 1.00)
Sugar intake infancy, servings/day	Reference	0.98 (0.86, 1.08)	1.01 (0.91, 1.12)
Screen time at 10 years	Reference	..	..
<2 h/day	..	Reference category	Reference category
≥2 h/day	..	0.96 (0.74, 1.24)	1.21 (0.93, 1.58)
Exercise at 10 years	Reference	..	..
<2 h/day	..	Reference category	Reference category
≥2 h/day	..	1.02 (0.75, 1.31)	0.94 (0.70, 1.27)

Note: These values represent the Odds Ratios and their subsequent 95% Confidence Intervals of being in the group represented by the column, when the characteristic increases by one unit or compared to their reference category, while all other risk factors stayed the same, adjusting for maternal education and household income. These analyses were done on 50 imputed, pooled data sets.

SDS: standard deviation scores. None of the associations were significant after 0.05 FDR correction based on 24 hypotheses: Each childhood characteristic was tested between all 3 outcomes, with 8 exposures (childhood characteristics) in total, creating a total of 24 hypotheses to be tested.

### Childhood liver fat, MAFLD and cardio-metabolic outcomes

**Fig. 2:**
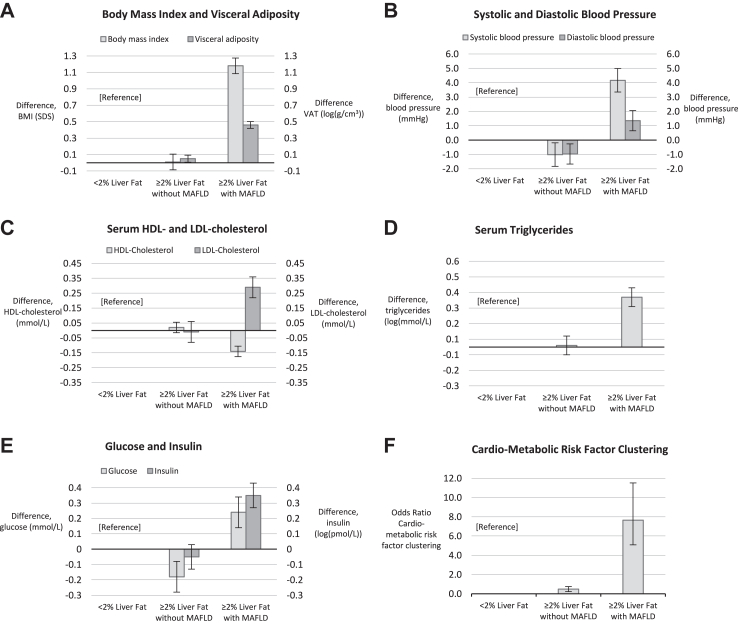
Liver fat, MAFLD and Cardio-metabolic Risk Factors. Effect estimates reflect the differences (95% Confidence Interval) in cardio-metabolic risk factors between children with ≥2% liver fat without and with Metabolic-Associated Fatty Liver Disease as compared to children with <2% liver fat.

Fig. 2 shows that, as compared to children with <2% liver fat, those with ≥2% liver fat and MAFLD had a higher BMI (difference 1.18 SDS, 95% CI 1.08, 1.28), higher visceral adiposity (difference 0.46 log (g/cm^3^), 95% CI 0.42, 0.50), higher systolic blood pressure (difference 4.17 mmHg, 95% CI 3.31, 5.02), higher diastolic blood pressure (difference 1.36 mmHg, 95% CI 0.66, 2.07), higher serum triglycerides (difference 0.32 log (mmol/L), 95% CI 0.26, 0.38), lower HDL-cholesterol (difference −0.14 mmol/L, 95% CI −0.17, −0.10), higher LDL-cholesterol (difference 0.29 mmol/L, 95% CI 0.22, 0.37), higher glucose (difference 0.24 mmol/L, 95% CI 0.14, 0.34), higher insulin (difference 0.35 log (pmol/L), 95% CI 0.27, 0.43), and increased odds of cardio-metabolic clustering (OR 7.65, 95% CI 5.04, 11.62). As compared to children with <2% liver fat, those with ≥2% liver fat without MAFLD had more visceral fat, lower blood pressure, and lower glucose concentrations (all FDR-adjusted p-values < 0.05). These findings were similar when children with ≥2% liver fat without MAFLD were compared to those with ≥2% liver fat with MAFLD (Supplementary Table S10). Results for the basic models are presented in Supplementary Table S11.

## Discussion

Results of this population-based study suggest a high prevalence of 25.5% of MAFLD among children in the general population, using a liver fat cut-off of ≥2%. None of the early childhood factors were associated with MAFLD after correction for socio-economic status and multiple testing, only Non-European ethnic background was nominally associated with increased odds of MAFLD. Compared to both children with <2% liver fat and ≥2% liver fat without MAFLD, children with MAFLD had an adverse cardiovascular risk factor profile and increased odds of cardio-metabolic risk factor clustering.

The MAFLD criteria have been defined to identify those individuals with excessive liver fat who also have signs of metabolic dysregulation.^6^ In our healthy population, 25.5% met these MAFLD criteria, whereas the prevalence among our participants with overweight or obesity was 72.5%. A previous meta-analyses estimated a global prevalence of around 33% among children and adolescents in the general ‘healthy’ population, and up to 67.4% among children with overweight or obesity in a clinical setting.^24^ The difference between our observed prevalence and those previously reported could be explained by differences in cut-off values, but also differences in imaging modalities. We have previously shown that a 2% liver fat fraction is already associated with an adverse cardio-metabolic risk profile at 10 years.^2^ Therefore, in the current study, we applied the cut-off of 2% liver fat fraction measured by MRI, instead of the most frequently used 5% liver fat cut-off. We did not observe a much higher prevalence as compared to other studies, although this what we initially expected due to the lower cut-off used. Our population, however, is most likely a more healthy group than previous studies and a healthy subsample of the general population, due to participation bias and a relatively large proportion of parents within our sample with a higher level of education. A previous meta-analysis estimated that roughly 19% of children with NAFLD are lean, and hypothesized that metabolic health is an important factor of fatty liver disease progression irrespective of BMI.^25^ Of all children with MAFLD in our study, 30.0% did not have overweight or obesity, and could be classified to fall into this ‘lean’ category. This finding highlights the role of overweight and obesity as a risk factors, but also suggest that a considerable number of lean children are affected by MAFLD as well.

None of the childhood risk factors observed in this study were associated with presence of MAFLD after correction for socio-economic factors and multiple testing. In our determinant analyses, breastfeeding, ≥2 h of screen-time per day and non-European descent appeared associated with MAFLD. However, after correcting for socio-economic factors, only non-European descent was nominally associated, and after multiple testing adjustment, this association did not persist. Despite this, we performed additional explorative analyses using more specific ethnic groups, which suggested that children of Cape Verdean, Turkish and Surinamese-Creole descent had increased risks compared to children of Dutch descent, but this association was only independent of socio-economic factors among children from Turkish descent. Ethnicity is a well-described risk factor for fatty liver disease in adults, with previously reported increased risks for those of Hispanic and Turkish descent, and a decreased risk for those of African descent.^26^ Previous reports suggested that the ethnic disparities in increased liver fat risk might be driven by a combination of genetic differences, socio-economic, and lifestyle differences.^27^ For example, specific variants in the PNPL3-gene that have been found to increase the risk of liver fat accumulation have been found to be more common in the Hispanic population in North America.^26^ As for other childhood risk factors, previous studies have suggested the beneficial effects of breastfeeding on a healthy childhood BMI and body fact distribution.^18^ As mentioned before, the associations of fewer months of breastfeeding and screen-time ≥2 h/day at 10 years with MAFLD at school age were explained by socio-economic factors. These results suggest that socio-economic disparities might be strong drivers behind certain childhood risk factors. However, as the outcome disparities between ethnicities could not be fully explained, although only nominally significant, and the ethnicities within the European pediatric population are underrepresented in previous studies regarding MAFLD, more follow up studies are needed focused on the ethnic disparities in risk for MAFLD within these populations.

Our results show that children with MAFLD do have more adverse cardio-metabolic outcomes and cardio-metabolic clustering than both those with <2% liver fat and those with ≥2% liver fat without MAFLD. These results are largely driven by de definition of MAFLD, which included some of the cardiovascular risk factors, as shown in Table 1. Our findings are in line with findings of studies in adult populations and clinically obese pediatric patients. Several studies reported that the MAFLD criteria better identify those individuals with increased cardiovascular risk and advanced liver disease.^28^^,^^29^ However, because our data are based on a cross-sectional analysis, further follow-up studies are needed to assess the associations of MAFLD in childhood with later life cardiovascular risk. A recent publication by the American Association for the Study of Liver Diseases (AASLD) suggested slight adjustments and renaming of the current MAFLD framework, namely Metabolic-Associated Steatotic Liver Disease (MASLD), as some experts in the field have raised concern over whether or not the name change was premature, and we still do not have a clear consensus on the pathophysiology, and therefore on biomarkers and therapies, of NAFLD.^30^ However, we would argue that the introduction of the criteria for MAFLD has created more awareness for hepatic steatosis in both adults and children in the scientific community, which will hopefully increase the frequency at which research into hepatic steatosis is currently being conducted. Furthermore, although there is little research on the long-term consequences of childhood MAFLD, the bidirectional relationships between fatty liver disease, type 2 diabetes, chronic kidney disease and cardiovascular events is well established in adults.^8^ Despite this, there are no concrete screening guidelines for MAFLD in the clinical setting, even though especially high risk patients with diabetes or cardiovascular disease could benefit from a multidisciplinary and holistic approach, through subsequent better risk stratification, early diagnosis and increased awareness by other care providers.^8^ Novel clinical trial designs applying such multidisciplinary approaches have already been proposed.^8^ Overall, our findings support the hypothesis that MAFLD criteria identify those with more adverse cardio-metabolic risk factor profiles. Considering the significant health burden of metabolic disease worldwide, future research should focus on implementing screening strategies, understanding the long-term consequences of pediatric MAFLD, and therapeutic strategies.

This study was embedded in an ongoing population-based prospective cohort study with detailed data-collection in a large sample size. MRI scans are considered a validated, accurate assessment tool of liver fat fraction.^14^ It is likely that selection bias was introduced due to children with missing a follow-up date in our study. However, if any selection bias, we would expect an underestimation of our effect estimates, because the children without follow up data tended to have overweight or obesity more frequently. Information on early-life risk factors were collected before and independent of the outcomes. Therefore, information bias in the risk factor analyses in unlikely. Additionally, the MAFLD criteria were adapted for this study, as mentioned, perhaps creating an overestimation of prevalence. Specifically, there is no previous evidence for the cut-off used for non-fasting serum glucose, as most research focuses on fasting serum glucose. This should be replicated in future studies, and these results should therefore be interpreted with caution. Some of the cardiovascular outcomes are included in the definition of MAFLD. The effect sizes of our associations of MAFLD with cardiovascular outcomes at the same age could therefore be inflated, and should be interpreted with keeping this in mind. Finally, residual confounding due to unmeasured or insufficient social or lifestyle factors is possible, as our results are based on an observational study design.

In this relatively healthy study sample, we observed a high prevalence of MAFLD, especially among children with overweight or obesity, suggesting that liver steatosis is a significant health burden in these children. We found no significant association between known NAFLD childhood risk factors and MAFLD, but child ethnicity was nominally significantly associated with MAFLD. Combined with further exploratory analyses, this suggests that ethnicity might be associated with increased odds of MAFLD from childhood onwards. Use of MAFLD criteria, rather than percentage of liver fat, might help to identify children at risk for adverse cardio-metabolic consequences of increased liver fat from school-age onwards. Further studies are needed to assess the long-term consequences of MAFLD in childhood on cardio-metabolic health in later life.

## Contributors

J.G. and V.W.V.J. were responsible for the study concept. J.G. and S.S. were responsible for the data collection and interpretation, statistical analysis, and manuscript draft. M.L.G, S.S., J.F., and V.W.V.J were responsible for the manuscript review. All authors were responsible for reading and approving the final manuscript.

## Data sharing statement

The data used in this study is derived from the Generation R Study based in Rotterdam. Individual researchers do not have the right to distribute this data from Generation R. For the purpose of verification/validation/replication/meta-analyses, the external researcher can contact our data managers (datamanagementgenr@erasmusmc.nl) and the Director of Generation R, Vincent Jaddoe (v.jaddoe@erasmusmc.nl). Data will be made available via these contact persons after a written agreement about the use of the data has been made via the Technology Transfer Office of the Erasmus Medical Center.

## Declaration of interests

We declare no competing interests.
