# Supplementary material for: Risk factors and cardio-metabolic outcomes associated with metabolic-associated fatty liver disease in childhood
Source: eClinicalMedicine. 2023 Oct 6;65:102248. doi: 10.1016/j.eclinm.2023.102248 (PMC10579278; doi:10.1016/j.eclinm.2023.102248)
Supplement: Supplementary Fig. S1 and Tables S1–S11 [file mmc1.docx]

# **Supplementary Tables and Figures**

# **Risk factors and Cardio-Metabolic Outcomes Associated with**

# **Metabolic Associated Fatty Liver Disease in Childhood**

Jasmin de Groot,^1,2^, Susana Santos,^1-4^, Madelon L. Geurtsen^1,2^,

Janine F. Felix^1,2^, Vincent W.V. Jaddoe^1,2^

1. The Generation R Study Group, Erasmus University Medical Center, Rotterdam, the Netherlands

2. Department of Pediatrics, Erasmus University Medical Center, Rotterdam, the Netherlands

3. EPIUnit, Instituto de Saúde Pública, Universidade do Porto, Porto, Portugal

4. Laboratório para a Investigação Integrativa e Translacional em Saúde Populacional (ITR), Universidade do Porto, Porto, Portugal

**Table of Contents**

**Supplementary Tables** page 1

Table 1 page 1

Table 2 page 2

Table 3 page 3

Table 4 page 4

Table 5 page 5

Table 6 page 6

Table 7 page 7

Table 8 page 8

Table 9 page 9

Table 10 page 10

Table 11 page 11

**Supplementary Figures**  page 12

Supplementary Figure 1A page 12

Supplementary Figure 1B page 13

**Supplementary Table 1. 90^th^ and 10^th^ percentiles used as cut-offs for metabolic criteria of MAFLD.**

|  | **10^th^ percentile** | **25^th^ percentile** | **75^th^ percentile** | **90^th^ percentile** |
| --- | --- | --- | --- | --- |
| Non-fasting serum Triglycerides (mmol/L) | n.a. | n.a. | 1.44 | 1.80 |
| Non-fasting serum HDL-cholesterol (mmol/L) | 1.09 | 1.23 | n.a. | n.a. |
| Systolic Blood Pressure (mmHg) | n.a. | n.a. | 109.3 | 113.3 |
| Diastolic Blood Pressure (mmHg) | n.a. | n.a. | 63.0 | 66.6 |
| Non-fasting serum glucose (mmol/L) | n.a. | n.a. | n.a. | 6.5 |
| Non-fasting serum insulin (pmol/L) | n.a. | n.a. | 310.4 | n.a. |
| Note: these percentiles are based on observed values within the participant population of n = 1910. Abbreviations: HDL: High-Density Lipoprotein. | | | | |

**Supplementary Table 2. Missing value proportions per variable.**

|  | **Percentage missing from total study population (%)** | |  |
| --- | --- | --- | --- |
| **Variables** | N = 1910 | |  |
| **Parental and pregnancy characteristics** |  | |  |
| Age mother at intake | 0.0 | |  |
| Maternal education | 7.2 | |  |
| Pre-pregnancy body mass index | 26.7 | |  |
| Multiparity | 3.3 | |  |
| Parental living situation | 7.7 | |  |
| Household income at 5 years | 16.1 | |  |
| Alcohol use during pregnancy | 20.5 | |  |
| Smoking during pregnancy | 13.1 | |  |
| Pregnancy complication |  | |  |
| Pre-eclampsia | 16.0 | |  |
| Gestational hypertension | 14.7 | |  |
| **Child characteristics** |  |  | |
| Female (%) | 0.0 | |  |
| Ethnicity | 2.0 | |  |
| Birth weight | 0.1 | |  |
| Gestational age at birth | 0.6 | |  |
| Breastfeeding | 30.6 | |  |
| Sugar intake at infancy | 39.6 | |  |
| Screen-time/day at 10 years | 23.1 | |  |
| Exercise/day at 10 years | 17.2 | |  |
| **Adiposity and Cardiovascular outcomes** |  | |  |
| Age assessment | 0.0 | |  |
| Liver fat fraction | 0.0 | |  |
| Body mass index | 0.0 | |  |
| Visceral adiposity | 0.0 | |  |
| Systolic blood pressure | 0.0 | |  |
| Diastolic blood pressure | 0.0 | |  |
| Glucose | 0.0 | |  |
| Insulin | 0.3 | |  |
| Triglycerides | 0.0 | |  |
| HDL-Cholesterol | 0.0 | |  |
| LDL-Cholesterol | 0.0 | |  |
| Note: Values are observed, not imputed data and represent means (SD), medians (95% range) or valid % (n) unless otherwise stated. The mean and standard deviation are given for all normally distributed continuous variables, and the median and 95% range for non-normally distributed variables. Abbreviations: HDL: High-Density Lipoprotein; LDL: Low-Density Lipoprotein | | |  |

**Supplementary Table 3. Subject characteristics according to liver fat category.**

|  | **< 2% Liver Fat** | | | | | **≥ 2% Liver Fat without Metabolic Associated Fatty Liver Disease** | | | **≥ 2% Liver Fat  with Metabolic Associated Fatty Liver Disease** | |  |
| --- | --- | --- | --- | --- | --- | --- | --- | --- | --- | --- | --- |
|  | N = 963 | | | | | N = 466 | | | N = 481 | |  |
| **Maternal pregnancy characteristics** | | | |  | | | |  | | |  |
| Age at intake (years) | 31.3 (4.6) | | | | | 31.7 (4.6) | | | 30.5 (5.1)* | |  |
| Higher education (%) | 58.3 (527) | | | | | 56.0 (242) | | | 43.0 (188)* | |  |
| Parents living together (%) | 89.1 (793) | | | | | 91.0 (393) | | | 89.1 (392) | |  |
| Household income at 5 years (%) |  | | | | |  | | |  | |  |
| < 2 000 euros | 14.9 (121) | | | | | 18.8 (74) | | | 26.3 (104)* | |  |
| 2 000 – 4 000 euros | 42.9 (349) | | | | | 43.1 (169) | | | 46.2 (183)* | |  |
| > 4 000 euros | 42.2 (343) | | | | | 38.3 (151) | | | 27.5 (109)* | |  |
| Multiparity (%) | 41.8 (390) | | | | | 47.2 (214) | | | 41.7 (192) | |  |
| Pre-pregnancy body mass index (kg/m^2^) | 22.1 (17.7, 33.5) | | | | | 22.3 (18.5, 32.4) | | | 23.9 (18.5, 38.3)* | |  |
| Any alcohol use during pregnancy (% yes) | 63.6 (491) | | | | | 61.5 (225) | | | 48.7 (185)* | |  |
| Any smoking during pregnancy (% yes) | 20.0 (170) | | | | | 20.9 (84) | | | 24.4 (100) | |  |
| Pre-eclampsia (% yes) | 1.1 (9) | | | | | 1.3 (5) | | | 3.9 (16)* | |  |
| Gestational hypertension (% yes) | 3.6 (30) | | | | | 3.2 (13) | | | 3.2 (13) | |  |
| **Child characteristics** |  |  |  | |  | |  | | |  | |
| Female (%) | 51.0 (491) | | | | | 51.3 (239) | | | 53.2 (256) | |  |
| Ethnicity (%) |  | | | | |  | | |  | |  |
| Dutch/European | 72.9 (690) | | | | | 71.3 (326) | | | 59.0 (276)* | |  |
| Non-European | 27.1 (256) | | | | | 28.7 (131) | | | 41.0 (192)* | |  |
| Birth weight (grams) | 3476 (539) | | | | | 3484 (571) | | | 3430 (564) | |  |
| Gestational age at birth (weeks) | 40.1 (36.3, 42.4) | | | | | 40.1 (35.8, 42.1) | | | 40.1 (35.6, 42.1) | |  |
| Preterm birth (%) | 3.2 (31) | | | | | 4.5 (21) | | | 4.6 (22) | |  |
| Breastfeeding duration (months) | 5.0 (0.0, 12.0) | | | | | 3.5 (0.0, 12.0) | | | 3.5 (0.0, 12.0)* | |  |
| Sugar intake at infancy (servings/day) | 1.57 (0.09-4.92) | | | | | 1.33 (0.09, 4.67) | | | 1.73 (0.00, 5.14) | |  |
| Less than 2 hours of screen-time/day at 10 years (%) | 51.6 (386) | | | | | 51.1 (191) | | | 41.5 (144)* | |  |
| More than 2 hours of exercise/day at 10 years (%) | 23.3 (191) | | | | | 23.1 (90) | | | 20.9 (78) | |  |
| **Adiposity and Cardiovascular outcomes** | | | | | | | | | | |  |
| Age at assessment (years) | 9.9 (9.5, 11.8) | | | | | 10.0 (9.4, 11.9) | | | 10.0 (9.5, 11.9) | |  |
| Liver fat fraction (percentage) | 1.69 (1.15, 1.99) | | | | | 2.31 (2.02, 4.32)* | | | 2.66 (2.02, 8.13)* | |  |
| Body mass index, (SDS) | -0.01 (0.91) | | | | | 0.02 (0.66)* | | | 1.28 (0.93)* | |  |
| Overweight (%) | 11.2 (108) | | | | | 0.0 (0) | | | 50.5 (243)* | |  |
| Obesity (%) | 2.1 (20) | | | | | 0.0 (0) | | | 19.5 (94)* | |  |
| Visceral adiposity (g/cm^3^) | 110.4 (54.3, 246.7) | | | | | 118.3 (57.7, 213.3)* | | | 187.4 (75.8, 394.0)* | |  |
| Systolic blood pressure (mmHg) | 102.2 (7.5) | | | | | 101.3 (6.9) | | | 106.9 (8.1)* | |  |
| Diastolic blood pressure (mmHg) | 58.3 (6.4) | | | | | 57.4 (5.8)* | | | 60.2 (6.6)* | |  |
| Glucose (mmol/L) | 5.26 (0.94) | | | | | 5.07 (0.74)* | | | 5.49 (1.01)* | |  |
| Insulin (pmol/L) | 164 (34, 572) | | | | | 160 (33, 513)* | | | 237 (51, 819)* | |  |
| Triglycerides (mmol/L) | 0.88 (0.40, 2.32) | | | | | 0.93 (0.43, 1.96) | | | 1.17 (0.44, 3.03)* | |  |
| HDL-Cholesterol (mmol/L) | 1.53 (0.33) | | | | | 1.55 (0.32) | | | 1.38 (0.34)* | |  |
| LDL-Cholesterol (mmol/L) | 2.74 (0.62) | | | | | 2.73 (0.60) | | | 3.05 (0.70)* | |  |
| Ratio Triglycerides:HDL | 0.59 (0.21, 2.06) | | | | | 0.61 (0.43, 1.96)* | | | 0.82 (0.25, 3.02)* | |  |
| Note: Values are observed, not imputed data and represent means (SD), medians (95% range) or valid % (n) unless otherwise stated. The mean and standard deviation are given for all normally distributed continuous variables, and the median and 95% range for non-normally distributed variables. *significantly different from those with < 2% Liver Fat ( p<0.05), determined using ANOVA or Chi-square tests. Abbreviations: SDS: Standard Deviation Score; HDL: High-Density Lipoprotein; LDL: Low-Density Lipoprotein. | | | | | | | | | | |  |

**Supplementary Table. Characteristics of MAFLD groups using 2% and 5% cut-off.**

|  | **≥ 2% Liver fat and Metabolic-Associated Fatty Liver Disease** | | **≥ 5% Liver fat and Metabolic-Associated Fatty Liver Disease** | |
| --- | --- | --- | --- | --- |
|  | N = 481 | | N = 42 | |
| **Parental and pregnancy characteristics** |  |  | | |
| Age mother at intake (years) | 30.6 (5.0) | | 30.3 (5.5) | |
| Maternal higher education (%) | 43.0 (188) | | 40.0 (16) | |
| Parents living together (%) | 89.1 (392) | | 87.8 (36) | |
| Household income at 5 years (%) |  | |  | |
| < 2 000 euros | 26.3 (104) | | 41.2 (14) | |
| 2 000 – 4 000 euros | 46.2 (183) | | 41.2 (14) | |
| > 4 000 euros | 27.5 (109) | | 17.6 (6) | |
| Multiparity (%) | 41.7 (192) | | 46.3 (19) | |
| Pre-pregnancy body mass index (kg/m^2^) | 23.9 (18.6, 38.2) | | 24.6 (19.9, 38.6) | |
| Alcohol use during pregnancy (% yes) | 48.7 (185) | | 41.4 (11) | |
| Smoking during pregnancy (% yes) | 24.4 (100) | | 25.0 (10) | |
| Pre-eclampsia (% yes) | 3.9 (16) | | 2.8 (1) | |
| Gestational hypertension (% yes) | 3.2 (13) | | 2.8 (1) | |
| **Child characteristics** |  |  | |  |
| Female (%) | 53.2 (256) | | 52.4 (22) | |
| Ethnicity (%) |  | |  | |
| Dutch/European | 59.0 (276) | | 54.8 (23) | |
| Non-European | 41.0 (192) | | 45.2 (19) | |
| Birth weight (grams) | 3433 (562) | | 3330 (635) | |
| Gestational age at birth (weeks) | 40.1 (35.7, 42.1) | | 39.9 (30.1, 42.5) | |
| Preterm birth (%) | 4.6 (22) | | 7.1 (3) | |
| Breastfeeding (months) | 3.5 (0.0, 12.0) | | 2.5 (0.0, 12.0) | |
| Sugar intake at infancy (servings/day) | 1.73 (0.00, 5.14) | | 2.20 (0.0, 4.6) | |
| Less than 2 hours of screen-time/day at 10 years (%) | 41.5 (144) | | 32.3 (10) | |
| More than 2 hours of exercise/day at 10 years (%) | 20.9 (78) | | 12.5 (4) | |
| **Adiposity and Cardiovascular outcomes** | | | | |
| Age assessment (years) | 10.0 (9.5, 11.9) | | 10.0 (9.6, 11.5) | |
| Liver fat fraction (percentage) | 2.66 (2.02, 8.06) | | 6.48 (5.09, 18.40) | |
| Body mass index, (SDS) | 1.28 (0.93)* | | 1.84 (0.81) | |
| Overweight (%) | 50.5 (243) | | 38.1 (16) | |
| Obesity (%) | 19.5 (94) | | 47.6 (20) | |
| Visceral adiposity (g/cm^3^) | 187.4 (75.8, 394.0) | | 258.9 (92.8, 462.4) | |
| Systolic blood pressure (mmHg) | 106.9 (8.1) | | 108.5 (9.3) | |
| Diastolic blood pressure (mmHg) | 60.2 (6.6) | | 61.4 (6.3) | |
| Glucose (mmol/L) | 5.49 (1.01) | | 5.34 (0.72) | |
| Insulin (pmol/L) | 237 (51, 819) | | 262 (43, 887) | |
| Triglycerides (mmol/L) | 1.17 (0.44, 3.03) | | 1.34 (0.49, 3.58) | |
| HDL-Cholesterol (mmol/L) | 1.38 (0.34) | | 1.34 (0.35) | |
| LDL-Cholesterol (mmol/L) | 3.05 (0.70) | | 3.31 (0.86) | |
| Ratio Triglycerides:HDL | 0.82 (0.44, 3.03) | | 1.07 (0.26, 7.09) | |
| Note: Values are observed, not imputed data and represent means (SD), medians (95% range) or valid % (n) unless otherwise stated. The mean and standard deviation are given for all normally distributed continuous variables, and the median and 95% range for non-normally distributed variables. Abbreviations: MAFLD: Metabolic-Associated Fatty Liver Disease; HDL: High-Density Lipoprotein; LDL: Low-Density Lipoprotein. | | | | |

**Supplementary Table 5. Subject characteristics of participants and non-participants.**

|  | **Participants** | | **Non-participants** | **p-value** |
| --- | --- | --- | --- | --- |
|  | N = 1,910 | | N = 2,223 |  |
| **Parental and pregnancy characteristics** |  |  | | |
| Age mother at intake (years) | 31.2 (4.7) | | 31.0 (5.1) | 0.27 |
| Maternal higher education (%) | 54.0 (957) | | 49.0 (985) | 0.00* |
| Married/Living together (%) | 89.6 (1578) | | 85.3 (1605) | 0.01* |
| Household income at 5 years (%) |  | |  | 0.02* |
| < 2 000 euros | 18.7 (299) | | 22.5 (403) | .. |
| 2 000 – 4 000 euros | 43.7 (701) | | 41.8 (747) | .. |
| > 4 000 euros | 37.6 (603) | | 35.7 (635) | .. |
| Multiparity (%) | 43.1 (796) | | 41.8 (895) | 0.41 |
| Pre-pregnancy body mass index (kg/m^2^) | 22.6 (18.1, 35.2) | | 22.6 (18.1, 34.6) | 0.32 |
| Alcohol use during pregnancy (% yes) | 59.4 (901) | | 54.5 (929) | 0.01* |
| Smoking during pregnancy (% yes) | 21.3 (354) | | 24.2 (475) | 0.03* |
| Pre-eclampsia (yes) | 3.4 (56) | | 2.2 (39) | 0.53 |
| Gestational hypertension (yes) | 31.2 (4.7) | | 4.3 (78) | 0.21 |
| **Child characteristics** |  |  | |  |
| Female (%) | 51.6 (986) | | 49.2 (1093) | 0.12 |
| Ethnicity (%) |  | |  | 0.05* |
| Dutch/European | 69.0 (1292) | | 65.8 (1433) | .. |
| Non-European | 31.0 (579) | | 34.2 (746) | .. |
| Birth weight (grams) | 3475 (553) | | 3369 (583) | 0.00* |
| Gestational age at birth (weeks) | 40.1 (35.9, 42.3) | | 40.1 (35.0, 42.3) | 0.01* |
| Preterm birth (%) | 3.9 (74) | | 7.5 (167) | 0.00* |
| Breastfeeding (months) | 3.5 (0.0, 12.0) | | 3.5 (0.0, 12.0) | 0.84 |
| Sugar intake at infancy (servings/day) | 1.57 (0.4, 4.8) | | 1.65 (0.06, 4.65) | 0.79 |
| Less than 2 hours of screen-time/day at 10 years (%) | 49.1 (721) | | 44.1 (917) | 0.01* |
| More than 2 hours of exercise/day at 10 years (%) | 22.7 (359) | | 22.2 (398) | 0.74 |
| **Adiposity and Cardiovascular outcomes** | | | | |
| Age assessment (years) | 10.0 (9.5, 11.9) | | 9.9 (9.5, 11.6) | 0.00* |
| Liver fat fraction (percentage) | 1.99 (1.23, 4.93) | | 1.99 (1.21-5.57) | 0.05* |
| Body mass index, (non-standardized) kg/m^2^ | 16.9 (14.1-23.9) | | 16.9 (14.0-25.0) | 0.12 |
| Visceral adiposity (g/cm^3^) | 125.0 (56.7, 321.7) | | 130.0 (60.3, 360.4) | 0.08 |
| Systolic blood pressure (mmHg) | 103.2 (7.8) | | 103.3 (8.2) | 0.45 |
| Diastolic blood pressure (mmHg) | 58.5 (6.4) | | 58.8 (6.5) | 0.26 |
| Glucose (mmol/L) | 5.27 (0.93) | | 5.21 (0.98) | 0.08 |
| Triglycerides (mmol/L) | 0.95 (0.41, 2.60) | | 0.98 (0.39, 2.62) | 0.39 |
| HDL-Cholesterol (mmol/L) | 1.50 (0.34) | | 1.49 (0.34) | 0.45 |
| LDL-Cholesterol (mmol/L) | 2.81 (0.65) | | 2.81 (0.72) | 0.75 |
| Ratio Triglycerides:HDL | 0.64 (0.22, 2.36) | | 0.66 (0.21, 2.36) | 0.67 |
| Note: Values are observed, not imputed data and represent means (SD), medians (95% range) or valid % (n) unless otherwise stated. The mean and standard deviation are given for all normally distributed continuous variables, and the median and 95% range for non-normally distributed variables. *significantly different (p<0.05), determined using ANOVA. Abbreviations: HDL: High-Density Lipoprotein; LDL: Low-Density Lipoprotein. | | | | |

**Supplementary Table 6. Childhood risk factors for increased liver fat and MAFLD, basic models.**

|  | | ≥ **2% Liver Fat without Metabolic-Associated Fatty Liver Disease**  **versus   < 2% Liver Fat** | ≥ **2% Liver Fat with Metabolic-Associated Fatty Liver Disease**  **versus  < 2% Liver Fat** | ≥ **2 % Liver Fat with Metabolic-Associated Fatty Liver Disease**  **versus** ≥ **2% Liver Fat without Metabolic-Associated Fatty Liver Disease** |
| --- | --- | --- | --- | --- |
|  | | **Odds Ratio (95% Confidence Interval)** | | |
| **Childhood characteristics** | |  |  |  |
| Sex | | .. | .. | .. |
| Male | | Reference category | Reference category | Reference category |
| Female | | 1.02 (0.82, 1.27) | 1.10 (0.88, 1.37) | 0.93 (0.71, 1.20) |
| Ethnicity | | .. | .. | .. |
| European | Reference category | Reference category | Reference category |  |
| Non-European | 1.09 (0.85, 1.39) | 1.83 (1.4~~6~~5, 2.31) | 1.69 (1.29, 2.22) |  |
| Birth weight, SDS | | 1.03 (0.92, 1.14) | 0.95 (0.85, 1.06) | 0.93 (0.82, 1.05) |
| Gestational age, weeks | | 1.00 (0.93, 1.07) | 0.96 (0.90, 1.02) | 0.96 (0.89, 1.04) |
| Breastfeeding, months | | 0.98 (0.95, 1.02) | 0.95 (0.92, 0.99) | 0.97 (0.93, 1.01) |
| Sugar intake infancy, servings/day | | 0.99 (0.89, 1.10) | 1.06 (0.96, 1.18) | 1.07 (0.95, 1.21) |
| Screen time at 10 years | | .. | .. | .. |
| < 2 hours/day | Reference category | Reference category | Reference category |  |
| ≥ 2 hours/day | 1.02 (0.80, 1.29) | 1.50 (1.19, 1.96) | 1.50 (1.13, 2.01) |  |
| Exercise at 10 years | | .. | .. | .. |
| < 2 hours/day | Reference category | Reference category | Reference category |  |
| ≥ 2 hours/day | 1.01 (0.76, 1.33) | 0.93 (0.69, 1.24) | 0.93 (0.66, 1.30) |  |
| Note: These values represent the Odds Ratios and their subsequent 95% Confidence Intervals of being in the first group compared to the second group according to the title of the column, when the characteristic increases by one unit or compared to their reference category, adjusted only for age at measurement. These analyses were done on 50 imputed, pooled data sets. Abbreviations: SDS, standard deviation score. | | | | |
|  | |  |  |  |

**Supplementary Table 7. Childhood risk factors for increased liver fat and MAFLD, determinant model.**

|  | **< 2% Liver Fat** | | **≥ 2% Liver Fat without Metabolic-Associated Fatty Liver Disease** | | **≥2% Liver Fat with Metabolic-Associated Fatty Liver Disease** |
| --- | --- | --- | --- | --- | --- |
|  | **Odds Ratio (95% Confidence Interval)** | | | | |
| **Childhood characteristics** |  |  | |  | |
| Sex | Reference | | .. | | .. |
| Male | .. | | Reference category | | Reference category |
| Female | .. | | 1.02 (0.81, 1.28) | | 1.14 (0.91, 1.43) |
| Ethnicity | Reference | | .. | | .. |
| European | .. | | Reference category | | Reference category |
| Non-European | .. | | 1.10 (0.85, 1.43) | | 1.68 (1.31, 2.16) |
| Birth weight, SDS | Reference | | 1.04 (0.93, 1.16) | | 1.00 (0.89, 1.11) |
| Gestational age, weeks | Reference | | 1.00 (0.94, 1.07) | | 0.98 (0.92, 1.05) |
| Breastfeeding, months | Reference | | 0.98 (0.95, 1.02) | | 0.96 (0.93, 0.99) |
| Sugar intake infancy, servings/day | Reference | | 0.99 (0.89, 1.10) | | 1.03 (0.93, 1.14) |
| Screen time at 10 years | Reference | | .. | | .. |
| < 2 hours/day | .. | | Reference category | | Reference category |
| ≥ 2 hours/day | .. | | 1.01 (0.79, 1.31) | | 1.31 (1.01, 1.70) |
| Exercise at 10 years | Reference | | .. | | .. |
| < 2 hours/day | .. | | Reference category | | Reference category |
| ≥ 2 hours/day | .. | | 1.01 (0.76, 1.33) | | 0.94 (0.70, 1.26) |
| Note: These values represent the Odds Ratios and their subsequent 95% Confidence Intervals of being in the group represented by the column, when the characteristic increases by one unit or compared to their reference category, while all other childhood characteristics stayed the same. These analyses were done on 50 imputed, pooled data sets. Abbreviations: SDS: Standard Deviation Scores. | | | | | |

**Supplementary Table 8. Childhood risk factors for increased liver fat and MAFLD, exploratory analysis with specific ethnicity groups.**

|  | **< 2% liver fat** | | **≥ 2% Liver Fat without Metabolic-Associated Fatty Liver Disease** | | **≥2% liver fat with Metabolic-Associated Fatty Liver Disease** |
| --- | --- | --- | --- | --- | --- |
|  | **Odds Ratio (95% Confidence Interval)** | | | | |
| **Childhood characteristics** |  |  | |  | |
| Sex | Reference | | .. | | .. |
| Male | .. | | Reference category | | Reference category |
| Female | .. | | 1.02 (0.80, 1.30) | | 1.18 (0.92, 1.51) |
| Ethnicity | Reference | | .. | | .. |
| Dutch/European (n=1292) | .. | | Reference category | | Reference category |
| Cape Verdean (n=54) | .. | | 1.59 (0.75, 3.36) | | 1.98 (0.99, 3.95) |
| Dutch Antillean (n=55) | .. | | 0.79 (0.37, 1.67) | | 1.10 (0.57, 2.11) |
| Moroccan (n=87) | .. | | 0.60 (0.32, 1.14) | | 0.92 (0.53, 1.60) |
| Surinamese-Creole (n=46) | .. | | 0.95 (0.42, 2.15) | | 1.66 (0.83, 3.29) |
| Surinamese-Hindustani (n=55) | .. | | 1.22 (0.62, 2.38) | | 0.84 (0.41, 1.72) |
| Turkish (n=95) | .. | | 1.16 (0.63, 2.14) | | 2.50 (1.50, 4.16) |
| Birth weight, SDS | Reference | | 1.04 (0.92, 1.17) | | 1.02 (0.90, 1.15) |
| Gestational age, weeks | Reference | | 1.01 (0.94, 1.09) | | 0.99 (0.92, 1.06) |
| Breastfeeding, months | Reference | | 0.98 (0.95, 1.02) | | 0.96 (0.92, 0.99) |
| Sugar intake infancy, servings/day | Reference | | 0.98 (0.87, 1.11) | | 1.03 (0.92, 1.16) |
| Screen time at 10 years | Reference | | .. | | .. |
| < 2 hours/day | .. | | Reference category | | Reference category |
| ≥ 2 hours/day | .. | | 0.97 (0.74, 1.27) | | 1.27 (0.96, 1.69) |
| Exercise at 10 years | Reference | | .. | | .. |
| < 2 hours/day | .. | | Reference category | | Reference category |
| ≥ 2 hours/day | .. | | 0.92 (0.68, 1.24) | | 0.87 (0.63, 1.20) |
| Note: These values represent the Odds Ratios and their subsequent 95% Confidence Intervals of being in the group represented by the column, when the characteristic increases by one unit or compared to their reference category while all other characteristics stay the same, controlling for maternal education and household income. These analyses were done on 50 imputed, pooled data sets. Abbreviations: SDS: Standard Deviation Scores. | | | | | |

**Supplementary Table 9. Childhood risk factors for increased liver fat, 2% liver fat without MAFLD compared to MAFLD, determinant model and full model.**

|  | ≥ **2% Liver Fat without MAFLD** | ≥ **2% Liver Fat with Metabolic-Associated Fatty Liver Disease,**  **Determinant model** | | ≥ **2% Liver Fat with Metabolic-Associated Fatty Liver Disease,**  **Full model^a^** |
| --- | --- | --- | --- | --- |
|  | **Odds Ratio (95% Confidence Interval)** | | | |
| **Childhood characteristics** |  |  | |  |
| Sex | Reference | | .. | .. |
| Male | .. | | Reference category | Reference category |
| Female | .. | | 1.13 (0.87, 1.47) | 1.13 (0.87, 1.47) |
| Ethnicity | .. | | .. | .. |
| European | Reference | | Reference category | Reference category |
| Non-European | .. | | 1.54 (1.15, 2.06) | 1.38 (1.01, 1.90) |
| Birthweight, SDS | Reference | | 0.97 (0.85, 1.10) | 0.98 (0.86, 1.11) |
| Gestational age, weeks | Reference | | 0.98 (0.91, 1.06) | 0.9~~8~~ (0.91, 1.06) |
| Breastfeeding, months | Reference | | 0.98 (0.94, 1.02) | 0.98 (0.94, 1.02) |
| Sugar intake infancy, servings/day | Reference | | 1.05 (0.93, 1.18) | 1.03 (0.92, 1.17) |
| Screen time at 10 years | Reference | | .. | .. |
| < 2 hours/day | .. | | Reference category | Reference category |
| ≥ 2 hours/day | .. | | 1.32 (0.97, 1.80) | 1.24 (0.90, 1.71) |
| Exercise at 10 years | Reference | | .. | .. |
| < 2 hours/day | .. | | Reference category | Reference category |
| ≥ 2 hours/day | .. | | 0.91 (0.64, 1.29) | 0.91 (0.64,1.29) |
| Note: These values represent the Odds Ratios and their subsequent 95% Confidence Intervals of being in the group represented by the column, when the characteristic increases by one unit or compared to their reference category, while all other childhood characteristics stayed the same. ^a^The full model additionally adjusted for maternal education and household income. These analyses were done on 50 imputed, pooled data sets. Abbreviations: SDS: Standard Deviation Scores. | | | | |

**Supplementary Table 10. Difference of cardio-metabolic outcomes between children with more than 2% liver fat without MAFLD and children with MAFLD.**

|  |  | |  |
| --- | --- | --- | --- |
|  | **≥ 2% Liver Fat without Metabolic-Associated Fatty Liver Disease** | **≥ 2 % Liver Fat with Metabolic-Associated Fatty Liver Disease** | |
|  | **Difference (95% Confidence Interval) in Cardio-metabolic outcome** | | |
| Body Mass Index, SDS^a^ | Reference | 1.16 (1.06, 1.27) | |
| Visceral Adiposity, log(g/cm^3^)^a^ | Reference | 0.41 (0.36, 0.46) | |
| Systolic blood pressure, mmHg^a^ | Reference | 5.24 (4.24, 6.24) | |
| Diastolic blood pressure, mmHg^a^ | Reference | 2.37 (1.55, 3.18) | |
| Serum Triglycerides, log(mmol/L)^a^ | Reference | 0.32 (0.24, 0.39) | |
| Serum HDL-cholesterol, mmol/L^a^ | Reference | -0.15 (-0.20, -0.11) | |
| Serum Glucose, mmol/L^a^ | Reference | 0.45 (0.33, 0.57) | |
| Serum Insulin, log(pmol/L)^a^ | Reference | 0.41 (0.32, 0.50) | |
| Serum LDL cholesterol, mmol/L^a^ | Reference | 0.30 (0.21, 0.39) | |
|  | **Odds Ratio (95% Confidence Interval) for Cardio-metabolic Clustering** | | |
| Clustering^b^ | Reference | 15.84 (7.57, 33.14) | |
| Note: ^a^These values represent the effect estimates and their corresponding 95% Confidence intervals, based on 50 imputed, pooled datasets. The effect estimate represents the change in cardio-metabolic outcome compared to the reference group, controlled for sex, ethnicity, maternal age, maternal education, pre-pregnancy BMI, parental living situation, household income, any alcohol use during pregnancy, any smoking during pregnancy, pregnancy complications, gestational age at birth, breastfeeding months, sugar intake in infancy, hours of exercise per day and screen-time per day at 10 years. ^b^These values represent Odds Rations with their 95% Confidence intervals from logistic regression analysis, controlling for sex, ethnicity, maternal age, maternal education, pre-pregnancy BMI, parental living situation, household income, any alcohol use during pregnancy, any smoking during pregnancy, pregnancy complications, gestational age at birth, breastfeeding months, sugar intake in infancy, hours of exercise per day and screen-time per day at 10 years, and based on 10 imputed, pooled datasets. BMI Standard Deviation Scores are calculated based on the growth curves developed by de Onis, et al. (2007). Clustering is defined as having three or more out of the following four adverse risk factors: visceral fat mass above the 75^th^ percentile; systolic or diastolic blood pressure above the 75^th^ percentile; HDL cholesterol below the 25^th^ percentile or triglycerides above the 75^th^ percentile; and insulin above the 75^th^ percentile of our study. Abbreviations: SDS: Standard Deviation Scores; HDL: High-density Lipoprotein; LDL: Low-density Lipoprotein. | | | |

**Supplementary Table 11. Liver fat categories and cardio-metabolic outcomes, basic models.**

|  |  | |  | |
| --- | --- | --- | --- | --- |
|  | ≥ **2% Liver Fat without Metabolic-Associated Fatty Liver Disease**  **versus   < 2% Liver Fat** | ≥ **2% Liver Fat with Metabolic-Associated Fatty Liver Disease**  **versus  <2% Liver Fat** | | ≥ **2 % Liver Fat with Metabolic-Associated Fatty Liver Disease**  **versus** ≥ **2% Liver Fat without Metabolic-Associated Fatty Liver Disease** |
|  | **Difference (95% Confidence Interval) in cardio-metabolic outcome** | | | |
| Body Mass Index, SDS^a^ | 0.03 (-0.07, 0.12) | 1.29 (1.19, 1.38) | | 1.26 (1.16, 1.37) |
| Visceral Adiposity, log(g/cm^3^)^a^ | 0.05 (0.01, 0.09) | 0.48 (0.44, 0.52 | | 0.43 (0.38, 0.48) |
| Systolic Blood Pressure, mmHg^a^ | -0.91 (-1.74, -0.08) | 4.73 (3.90, 5.55) | | 5.63 (4.67, 6.59) |
| Diastolic Blood Pressure, mmHg^a^ | -0.89 (-1.59, 0.20) | 1.91 (1.22, 2.60) | | 2.80 (2.01, 3.59) |
| Serum Triglycerides, log(mmol/L)^a^ | 0.02 (-0.03, 0.07) | 0.27 (0.22, 0.32) | | 0.34 (0.27, 0.41) |
| Serum HDL cholesterol, mmol/L^a^ | 0.02 (-0.02, 0.05) | -0.15 (-0.18, -0.11) | | -0.16 (-0.21, -0.12) |
| Serum Glucose, mmol/L^a^ | -0.19 (-0.29, -0.09) | 0.23 (0.13, 0.33) | | 0.42 (0.31, 0.53) |
| Serum Insulin, log(pmol/L)^a^ | -0.06 (-0.14, 0.02) | 0.39 (0.31, 0.46) | | 0.45 (0.36, 0.54) |
| Serum LDL cholesterol, mmol/L^a^ | -0.01 (-0.08, 0.06) | 0.31 (0.24, 0.38) | | 0.32 (0.23, 0.40) |
|  | **Odds Ratio (95% Confidence Interval) for Cardio-metabolic Clustering** | | | |
| Clustering^b^ | 0.48 (0.22, 1.04) | 8.8~~0~~5 (5.92, 13.23) | | 18.44 (8.89, 38.26) |
| Note: ^a^These values represent the effect estimates and their corresponding 95% Confidence intervals, based on 50 imputed, pooled datasets. The effect estimate represents the change in cardio-metabolic outcome compared to the reference group, controlled for age and sex only. ^b^These values represent Odds Ratios with their 95% Confidence intervals from logistic regression analysis, controlled for age and sex only, based on 10 imputed, pooled datasets. BMI Standard Deviation Scores are calculated based on the growth curves developed by de Onis, et al. (2007). Clustering is defined as having three or more out of the following four adverse risk factors: visceral fat mass above the 75^th^ percentile; systolic or diastolic blood pressure above the 75^th^ percentile; HDL cholesterol below the 25^th^ percentile or triglycerides above the 75^th^ percentile; and insulin above the 75^th^ percentile of our study. Abbreviations: BMI: SDS: Standard Deviation Scores; HDL: High-density Lipoprotein; LDL: Low-density Lipoprotein. | | | | |

**Supplementary Figures**

**Supplementary Figure 1 A and B – 1A. Directed-Acyclic Graph showing the assumed relationships between childhood factors and MAFLD:** for this cross-sectional analyses the main association(s) of interest are indicated by the full arrows.

= possible confounding relationship

= main association(s) of interest

= precision variable (to reduce the standard errors)

Age at MRI scan

Breastfeeding
Sugar intake infancy
Screen-time school-age
Exercise school-age

Birth weight
Gestational age at birth

Education
Household income

Sex
Ethnicity

MAFLD

**1B. Directed-Acyclic Graph showing the assumed relationships between early life factors and the relationship between MAFLD and Cardio-metabolic risk factor clustering.**

= possible confounding relationship

= main association(s) of interest

= precision variable (to reduce the standard errors)

Parental

Age mother
Education
Pre-pregnancy BMI
Marital status
Household income
Parity

MAFLD

Age at MRI

Cardio-metabolic risk factor clustering

Childhood

Birth weight
Gestational age
Sex
Ethnicity
Breastfeeding
Sugar intake infancy
Screen-time school-age
Exercise school-age

Pregnancy

Alcohol use
Smoking
Pregnancy complications
